# Assessment of the impact of the new blister packaging of Biktarvy^®^ (B/F/TAF) on treatment satisfaction of people living with HIV

**DOI:** 10.1371/journal.pone.0313458

**Published:** 2025-12-16

**Authors:** João Paulo Cruz, Osvaldo Santos, Marisa Rodrigues, Raquel Morais, Teresa Borralho, Filipa Ferreira, Abigail Ferreira, João Albuquerque, Paulo Nogueira, Maria Pinto da Silva, Francisco Antunes

**Affiliations:** 1 Serviços Farmacêuticos da Unidade de Saúde Local Santa Maria, Lisboa, Portugal; 2 iMed.ULisboa – Institute for Medicine Research, Faculdade de Farmácia, Universidade de Lisboa, Lisboa, Portugal; 3 Instituto de Saúde Ambiental, Faculdade de Medicina, Universidade de Lisboa, Lisboa, Portugal; 4 Unbreakable Idea Research, Painho, Portugal; 5 BlueClinical – Investigação e Desenvolvimento em Saúde, Lda, Porto, Portugal; 6 Centro de Estatística e Aplicações, Faculdade de Ciências, Universidade de Lisboa, Lisboa, Portugal; 7 Gilead Sciences, Lda, Lisboa, Portugal; 8 Laboratório Associado TERRA, Universidade de Lisboa, Lisboa, Portugal; The University of Sydney, AUSTRALIA

## Abstract

**Background:**

Antiretroviral therapy (ART) is highly effective in people living with HIV (PLHIV), but its success depends on treatment satisfaction and adherence. A determinant of satisfaction regards how the medication is delivered to the patient, namely how it is contained (e.g., bottles, blisters, etc). A new packaging of Biktarvy^®^ has been introduced as a monthly blister, aiming to improve satisfaction, facilitate traceability of daily medication, portability, and discretion (reducing stigma associated with ART), and, ultimately, enhance adherence.

**Goals:**

The study’s objective was to assess the impact of changing the packaging of Biktarvy® (B/F/TAF) from a standard pill bottle to a monthly blister with a weekly calendar on therapy satisfaction. Additionally, the association between treatment satisfaction and selected patients’ characteristics (e.g., ART duration) was evaluated. A secondary goal was to characterize the association between the change of packaging on patient’s adherence.

**Methods:**

This is an observational longitudinal (retrospective and prospective) study with patients following ART for at least six months (ambulatory clinical management) recruited according to a non-probabilistic sequential sampling. Satisfaction was measured at two different moments: at baseline, HIVTSQs were used to assess satisfaction within the previous six months’ use of medication containers (bottles). Six months later, patients filled in the HIVTSQc to assess their perception of satisfaction change with the new packaging (blister). Adherence was assessed by pharmacy medication dispensing at the hospital.

**Results:**

The study enrolled 105 patients in two selected centers (102 patients completed the study). Patients were significantly more satisfied (HIVTSQc scores) with ART when using the new Biktarvy® blister pack package. Importantly, gains of ART satisfaction were higher among those less satisfied with the bottle packaging. No significant associations were found between HIVTSQc scores and sociodemographic or ART-related variables.

## Introduction

In the last decade, antiretroviral therapy (ART) has demonstrated high efficacy, resulting in higher than 90% viral suppression rates [1]. Therapeutic guidelines identify several regimens that allow adequate long-term suppression of viral load in people living with HIV infection (PLHIV), including single tablet coformulations [2].

The success of ART depends on treatment satisfaction and adherence, which are impacted by several factors, including medication simplicity and dosing convenience, patient perception of results, and side effects [3]. These factors must be considered when making treatment decisions in the clinical management of PLHIV [4]. For all these reasons, satisfaction has been considered relevant in the assessment and differentiation between therapeutic regimens, aiming to improve clinical outcomes, the well-being of PLHIV, and maximizing improvement of health status [5,6].

Medication packaging in blister packs, with a weekly calendar, has the potential to contribute to improving adherence to ART and optimizing outcomes in the treatment of PLHIV. In the scope of treatment of hypertension, patients with medication in blisters with weekly calendars renewed their prescription more frequently, had higher Medication Possession Ratio (MPR) and better control of blood pressure at 12 months [7]. Additionally, a meta-analysis of 52 studies (n = 22 858) involving patients with different diseases showed an increase in adherence from 63% to 71% when packaging was changed from traditional pill bottles to blister packs with a calendar [8].

Biktarvy® is indicated for the treatment of adults infected with human immunodeficiency virus type 1 (HIV-1) without present or past evidence of viral resistance to the integrase inhibitor class, emtricitabine or tenofovir. Currently, Biktarvy® new package contains the same number of units (30 tablets), but packaged in four blisters of seven tablets, with a weekly calendar, and one blister of two tablets.This new packaging may help improve patient adherence to therapy by making it easier to track daily medication intake. Its discreet and portable presentation also supports greater privacy, which can contribute to reducing the stigma often associated with ART. In Portugal, the units of Biktarvy® in bottles were completely replaced by blister packs by May 2022 [9].

This project aimed to estimate the impact of the new Biktarvy® packaging containing the same number of units (30 tablets), but packed in four blisters of seven tablets, with a weekly calendar, and one blister of two tablets, on patient’s satisfaction (main outcome) and adherence (secondary outcome) with ART.

## Materials and methods

### Research strategies

The study followed an observational retrospective/prospective design with PLHIV being followed in two hospitals in Portugal. Data collected referred to a period of ART of at least six months before the change of packaging (bottle; retrospective data) and six months of experience with the new packaging (blister; prospective data).

The study was conducted in accordance with the clinical study protocol (CSP), the International Council for Harmonization, Good Clinical Practices, and the Declaration of Helsinki, as well as the applicable European and Portuguese laws and regulations approved by the Ethics Committee of the Centro Académico de Medicina de Lisboa (reference number 226/22).

All patients signed a written informed consent form before entering the study. Each participant received a full explanation of the project goals, procedures, and implied tasks (and burden) from patients. Subjects were informed that their participation was voluntary and assured that they could abandon the study at any time without any prejudice. They also received a copy of the subject’s information and, after being fully clarified, signed the informed consent form.

### Sampling: Inclusion and exclusion criteria

The sample size was planned to enroll 100 PLHIV (including at least 20 women) on treatment with Biktarvy®, being over 18 years old, under ambulatory clinical management in two Portuguese hospitals for at least six months and accepting to participate voluntarily in the study.

Patients coming to the hospital pharmacy were included sequentially (non-probabilistic sampling), and the sample size was determined to allow an estimate of the patients with timely prescription delivery in the hospital pharmacy (before the time of prescription collection). Given the paired nature of the satisfaction measures (pre/post), a within-subjects design was considered for the calculation of the minimum sample size required. A moderate effect size (Cohen’s d = 0.5) was assumed as clinically meaningful for the HIVTSQ satisfaction score difference, based on similar studies evaluating patient-reported outcomes in ART packaging interventions [10].To detect this difference with a power of 80% (1-β = 0.80) and a significance level of 5% (α = 0.05, two-tailed), a minimum of 64 participants would be required for a paired comparison. Accounting for a potential dropout or incomplete data rate of 30%, a total sample of approximately 92 participants was considered necessary. To ensure adequate representation and buffer against unexpected exclusions, the study aimed to recruit at least 100 patients. The first patient visit occurred in August 2022, and the last patient visit occurred in December 2023.

### Data collection and instruments

Subjects’ data was directly collected on paper-based clinical reports forms (CRFs). Subsequently, data were transcribed into the study database by a data entry operator. Each patient was given a study reference number, which was archived in the clinical file.

Data collected included demographic information (age, gender, nationality, level of education), clinical and laboratory data (duration of ART, viral load, and CD4 T lymphocytes count). For assessing satisfaction with ART, two instruments have been used: the Portuguese version of the HIV Treatment Satisfaction Questionnaires version “s” (HIVTSQs), at baseline (retrospective assessment related to the previous six months), and the Portuguese version for the HIV Treatment Satisfaction Questionnaires version “c” (HIVTSQc), six months after starting the use of Biktarvy^®^ monthly blisters. HIVTSQs, the status version, which measures satisfaction with the current treatment, and HIVTSQc, the change version, which measures satisfaction with the change in treatment, have been developed by Woodcock & Bradley, with evidence of sound psychometric properties [10,11]. The HIVTSQs is composed of 10 items being answered in a seven-points Likert-type scale (varying from 0 = “very dissatisfied” to 6 = “very satisfied”). The HIVTSQs provides a global score of satisfaction as well as scores for two subdimensions: the HIVTSQs General Satisfaction/Clinical subscale and the HIVTSQs Lifestyle/Ease subscale; the HIVTSQc also includes a subscale: the HIVTSQc General Satisfaction/Clinical subscale.

Additionally, and for a better understanding of the factors of satisfaction with ART, patients were asked about their level of agreement with four statements, both at baseline and at follow-up: *The packaging of this medication allows me to easily check if I’ve taken my daily pill*, *This medicine comes in a package that lets me take the pill easily and without anyone noticing*, *This medicine’s packaging reminds me that I need to refill my prescription*, and *I don’t like the way this medicine is packaged*. Finally, patients reported their level of agreement with one more statement, at the follow-up moment: *I am more satisfied with this type of medication packaging than with the previous one, which was a plastic bottle*. Level of agreement was indicated through a five-points Likert scale ranging from *1* = *totally disagree* to *5* = *totally agree*.

**Fig 1:**
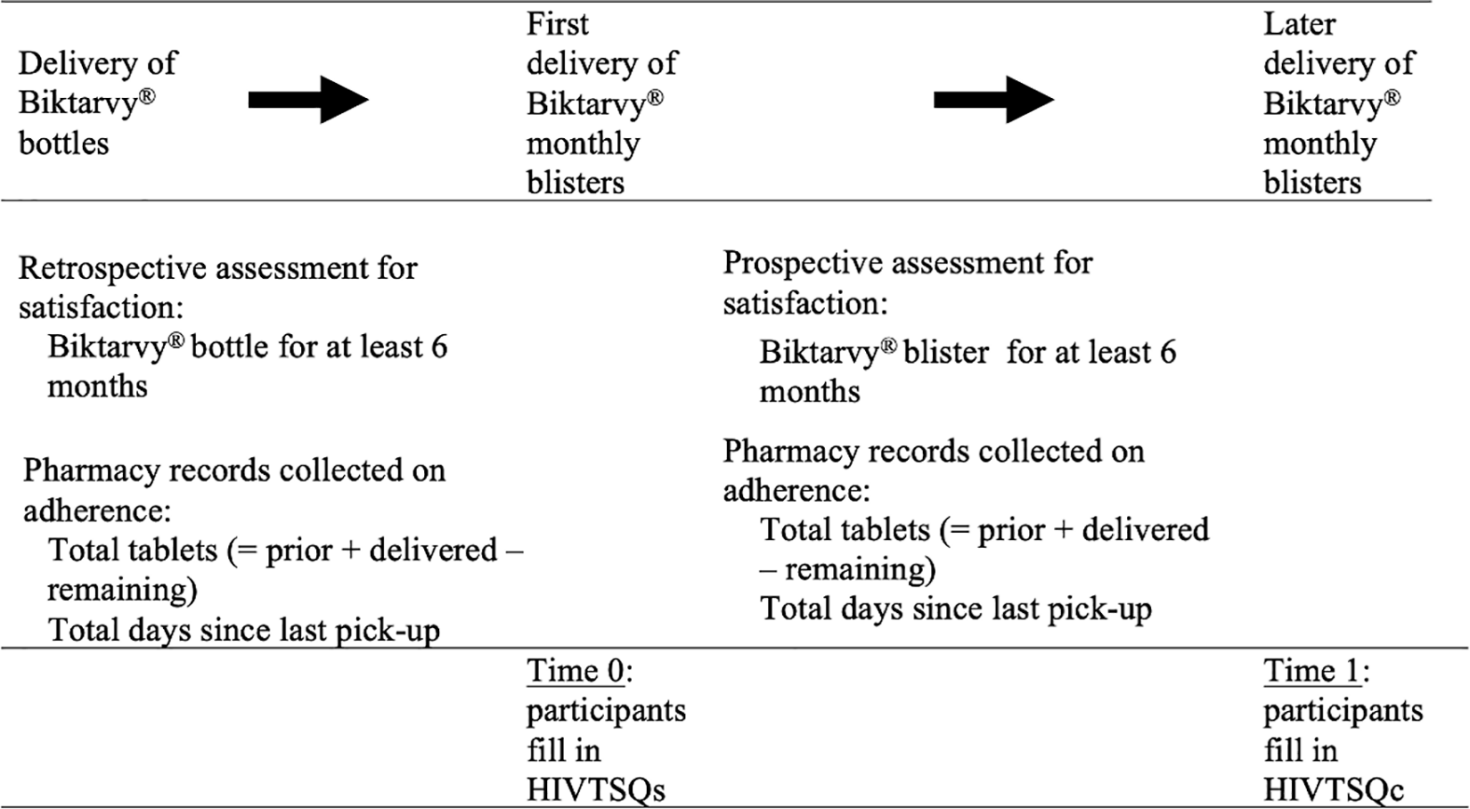
Study design diagram.

To assess the impact of the new Biktarvy® packaging on adherence, pharmacy medication dispensing was measured, more specifically, by registering the number of tablets supplied in the last delivery per number of days elapsed since the last delivery. Pharmacy medication dispensing data were collected by pharmacy records, and questionnaires were completed while patients waited in the hospital pharmacy (a representation of the study design is presented in Fig 1).

### Data analysis

The statistical analyses were conducted using R statistical software version 4.2.1. Descriptive statistics were used to analyze results: percentages and absolute frequencies to describe the categorical variables; minimum, maximum, interquartile range, means, medians, and standard deviation for the quantitative variables.

All 10 items from HIVTSQs were summed up to obtain a treatment satisfaction score (0–60), where higher scores represent greater satisfaction. The HIVTSQs General Satisfaction/ Clinical subscale includes the items 1, 2, 3, 9 and 10 (range: 0–30), and the HIVTSQs Lifestyle/Ease subscale includes the remaining items, namely the items 4, 5, 6, 7 and 8 (range: 0–30).

For the HIVTSQc, the sum of all items results in a treatment satisfaction (change) score (range: −30–30), where positive values are associated with improvement in satisfaction with treatment, the 0 score represents no change, and negative values indicate a deterioration in treatment satisfaction. For the HIVTSQc General Satisfaction/ Clinical subscale and HIVTSQc Lifestyle/Ease subscale, the same items as in HIVTSQs subscales were included (range: −15–15). Cronbach alpha was used to assess internal consistency for both HIVTSQs and HIVTSQc, and correlations were applied to assess the convergent validity between these two scales.

To evaluate the effect of switching the medication from a standard pill bottle to a monthly blister in patient satisfaction, a unilateral Wilcoxon signed rank test was applied (alternative hypothesis: pseudomedian > 0) to the score in the HIVTSQc scale and its subscales. Differences between independent samples in the distribution of these scores was tested using the Wilcoxon rank sum test.

Linear regression models have been performed to assess the predictive effect of baseline satisfaction on satisfaction change. The scores for HIVTSQc scale, HIVTSQc General Satisfaction subscale, HIVTSQs Clinical subscale, e HIVTSQc Lifestyle/Ease subscale were found as very skewed (left-skewed distribution). Therefore, their values were shifted and reversed for modeling purposes, as follows: *HIVTSQc scale* recoded = 61 + 1 – (*HIVTSQc scale *+ 31); *HIVTSQc General Satisfaction/ Clinical subscale* recoded = 31 + 1 – (*HIVTSQc General Satisfaction/ Clinical subscale *+ 16); *HIVTSQc Lifestyle/Ease subscale* recoded = 31 + 1 – (*HIVTSQc Lifestyle/Ease subscale *+ 16). Resulting scores were log-transformed and included in the linear regression models as dependent variables. The models included as predictors sex, age group, education, nationality, and antiretroviral therapy duration, adjusting for the treatment satisfaction score at baseline. When studying the predictive effect of treatment satisfaction at baseline (for HIVTSQs scale, HIVTSQs General Satisfaction/ Clinical subscale, and HIVTSQs Lifestyle/Ease subscale) on HIVTSQc, HIVTSQc General Satisfaction/ Clinical e HIVTSQc Lifestyle/Ease, simple models (no additional variables, in these equations) have been used. The predictors mentioned above were also included all-together in one model (M2). Estimates and 95% confidence intervals (CI), and p-values are presented.

## Results

One hundred and five PLHIV were enrolled in the study. Two were discontinued due to a physician’s decision to switch ART, and one had not completed six months of using Biktarvy^®^ in blisters by the end of the data collection process (therefore, not considered for assessing satisfaction with the blister packaging). Table 1 summarizes the demographic and clinical characteristics of participants at baseline.

**Table 1:** Sociodemographic and clinical characteristics of the participants.

Sociodemographic variables		
Sex, n (%)	n	105
	Female, n (%)	22 (21.0)
	Male, n (%)	83 (79.1)
Age	n	105
	Mean	43.35
	SD	11.69
	Mediana [Q1, Q3]	42.00 (34.00, 53.00)
	Minimum	21
	Maximum	79
Scholarship level, n (%)	n	105
	No formal education	2 (1.9)
	Primary (basic) education	27 (25.7)
	Secondary education	37 (35.2)
	Superior education	39 (37.1)
Nationality, n (%)	n	105
	Portuguese	59 (56.20)
	Non-Portuguese	46 (43.8)
Clinical variables		
Antiretroviral therapy duration, n (%)	n	105
	Less than 1 year	2 (1.9)
	1 - 5 years	53 (50.5)
	6 - 10 years	14 (13.3)
	More than 10 years	36 (34.3)
HIV viral load, n (%)	n	105
	Not detectable	83 (79.0)
	20 - 200 copies/mL	21 (20.0)
	201 - 1000 copies/mL	1 (1.0)
CD4 T lymphocytes count, n (%)	n	105
	< 350 cells/mm³	6 (5.7)
	351 - 500 cells/mm³	15 (14.3)
	> 500 cells/mm³	84 (80.0)

n: number of subjects; HIV: human immunodeficiency virus.

Out of the 105 participants, 21 (21.0%) were females. The mean age was 43.35 years (s.d:11.69; min 21, max 79 years old), 35.2% and 37.1% had secondary and superior education, respectively, and 43.8% were born outside Portugal. Only two participants had been following ART for less than one year. 79.0% had a non-detectable viral load, and 80.0% had a CD4T lymphocyte count higher than 500 cells/mm [3].

**Fig 2:**
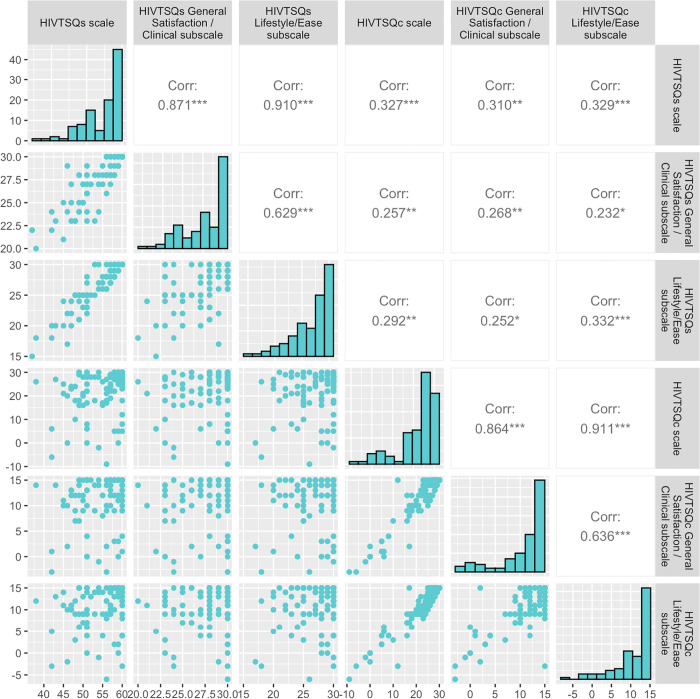
HIVTSQs (and subscales) and HIVTSQc total scores correlations (Spearman correlation).

The scales used for assessing satisfaction performed well psychometrically: the internal reliability coefficients for HIVTSQs and HIVTSQc were found to be good (alpha = .76 for HIVTSQs; alpha = .88 for HIVTSQc). The correlation between HIVTSQs and HIVTSQc was also significant (rho = .327) (Fig 2).

As Table 2 shows, the scores of HIVTSQs (at baseline) were not found to be associated with sociodemographic variables or the selected clinical indicators. When considering the six-month assessment (with blister package usage) with HIVTSQc, a significant positive satisfaction evolution was observed (also for the HIVTSQc subscale).

**Table 2:** Satisfaction (HIVTSQs and HIVTSQc scores) by sex, age group, education, nationality, and antiretroviral therapy duration.

	TOTAL^1^		Sex^2^			Age group^2^			Education^2^			Nationality^2^			Antiretroviral therapy duration^2^		
			Female	Male		Less than 50 years	50 years or more		Secondary education or less	Superior education		Portuguese	Non-Portuguese		Up to 5 years	6 years or more	
	N = 105	p^1^	n = 22	n = 83	p^2^	n = 69	n = 36	p^2^	n = 66	n = 39	p^2^	n = 59	n = 46	p^2^	n = 55	n = 50	p^2^
**HIVTSQs scale**																	
Mean (SD)	54.69 (5.52)	(a)	55.05 (6.37)	54.59 (5.31)	0.500	54.83 (5.82)	54.42 (4.96)	0.361	54.64 (6.10)	54.77 (4.46)	0.705	53.90 (5.70)	55.70 (5.18)	0.085	54.25 (4.99)	55.16 (6.07)	0.191
Median [Q1, Q3]	57.00 (51.00, 59.00)		57.50 (53.50, 59.75)	57.00 (51.00, 59.00)		57.00 (51.00, 60.00)	56.00 (51.00, 58.00)		57.00 (50.25, 59.00)	56.00 (51.00, 59.00)		56.00 (50.00, 59.00)	57.00 (54.00, 60.00)		56.00 (50.00, 59.00)	57.00 (53.25, 60.00)	
Minimum – Maximum	37.00 - 60.00		37.00 - 60.00	38.00 - 60.00		37.00 - 60.00	42.00 - 60.00		37.00 - 60.00	47.00 - 60.00		37.00 - 60.00	38.00 - 60.00		43.00 - 60.00	37.00 - 60.00	
**HIVTSQs General Satisfaction/ Clinical subscale**																	
Mean (SD)	27.78 (2.61)	(a)	27.59 (2.84)	27.83 (2.56)	0.844	28.07 (2.48)	27.22 (2.78)	0.116	27.68 (2.81)	27.95 (2.25)	0.899	27.31 (2.81)	28.39 (2.20)	0.098	27.71 (2.38)	27.86 (2.86)	0.292
Median [Q1, Q3]	29.00 (26.00, 30.00)		28.50 (24.75, 30.00)	29.00 (26.50, 30.00)		29.00 (27.00, 30.00)	28.00 (25.00, 30.00)		28.50 (27.00, 30.00)	29.00 (26.00, 30.00)		28.00 (25.00, 30.00)	29.00 (28.00, 30.00)		28.00 (27.00, 30.00)	29.50 (26.00, 30.00)	
Minimum – Maximum	20.00 - 30.00		22.00 - 30.00	20.00 - 30.00		20.00 - 30.00	21.00 - 30.00		20.00 - 30.00	23.00 - 30.00		21.00 - 30.00	20.00 - 30.00		23.00 - 30.00	20.00 - 30.00	
**HIVTSQs Lifestyle/Ease subscale**																	
Mean (SD)	26.90 (3.52)	(a)	27.45 (4.07)	26.76 (3.37)	0.199	26.75 (3.76)	27.19 (3.03)	0.890	26.95 (3.86)	26.82 (2.88)	0.508	26.59 (3.37)	27.30 (3.69)	0.074	26.55 (3.33)	27.30 (3.70)	0.145
Median [Q1, Q3]	28.00 (25.00, 30.00)		29.00 (28.00, 30.00)	28.00 (25.00, 30.00)		28.00 (25.00, 30.00)	28.00 (25.00, 30.00)		28.50 (25.00, 30.00)	27.00 (25.00, 30.00)		28.00 (25.00, 29.00)	29.00 (26.25, 30.00)		27.00 (25.00, 30.00)	28.50 (27.00, 30.00)	
Minimum – Maximum	15.00 - 30.00		15.00 - 30.00	17.00 - 30.00		15.00 - 30.00	20.00 - 30.00		15.00 - 30.00	20.00 - 30.00		15.00 - 30.00	17.00 - 30.00		17.00 - 30.00	15.00 - 30.00	
**HIVTSQc scale**																	
Mean (SD)	22.21 (9.14)	**< 0.001**	22.05 (10.27)	22.24 (8.93)	0.853	23.07 (7.96)	20.54 (10.99)	0.474	21.59 (9.00)	23.21 (9.40)	0.133	21.19 (10.23)	23.49 (7.46)	0.368	22.85 (8.06)	21.45 (10.31)	0.615
Median [Q1, Q3]	25.50 (18.25, 29.00)		26.00 (19.00, 28.50)	25.00 (18.50, 29.00)		26.00 (19.50, 28.50)	25.00 (18.00, 29.00)		25.00 (18.00, 28.00)	28.00 (22.00, 29.50)		25.00 (18.00, 28.00)	26.00 (19.00, 29.00)		26.00 (19.00, 29.00)	25.00 (18.50, 28.00)	
Minimum – Maximum	−9 - 30		−6 - 30	−9 - 30		−6 - 30	−9 - 30		−6 - 30	−9 - 30		−9 - 30	0 - 30		0 - 30	−9 - 30	
(NA)	3		3	0		2	1		3	0		2	1		0	3	
**HIVTSQc General Satisfaction/ Clinical subscale**																	
Mean (SD)	11.37 (4.74)	**< 0.001**	12.00 (5.11)	11.23 (4.67)	0.299	11.78 (4.21)	10.60 (5.60)	0.611	11.21 (4.65)	11.64 (4.93)	0.397	10.88 (5.10)	12.00 (4.20)	0.178	11.60 (3.99)	11.11 (5.52)	0.516
Median [Q1, Q3]	13.00 (10.00, 15.00)		14.00 (12.00, 15.00)	13.00 (10.00, 15.00)		13.00 (10.50, 15.00)	12.00 (9.00, 15.00)		12.00 (10.00, 15.00)	14.00 (11.00, 15.00)		12.00 (9.00, 15.00)	14.00 (10.00, 15.00)		13.00 (10.50, 15.00)	14.00 (9.50, 15.00)	
Minimum – Maximum	−3 - 15		−3 - 15	−3 - 15		−3 - 15	−3 - 15		−3 - 15	−3 - 15		−3 - 15	0 - 15		0 - 15	−3 - 15	
(NA)	3		3	0		2	1		3	0		2	1		0	3	
**HIVTSQc Lifestyle/Ease subscale**																	
Mean (SD)	10.83 (4.97)	**< 0.001**	10.05 (6.11)	11.01 (4.69)	0.621	11.30 (4.53)	9.94 (5.68)	0.262	10.38 (4.96)	11.56 (4.96)	0.094	10.32 (5.35)	11.49 (4.41)	0.375	11.25 (4.33)	10.34 (5.63)	0.501
Median [Q1, Q3]	13.00 (9.00, 15.00)		13.00 (8.50, 14.00)	13.00 (9.00, 15.00)		13.00 (9.00, 15.00)	12.00 (8.50, 14.00)		12.00 (9.00, 14.00)	14.00 (9.50, 15.00)		12.00 (9.00, 15.00)	13.00 (9.00, 15.00)		13.00 (9.00, 15.00)	12.00 (9.00, 14.50)	
Minimum – Maximum	−6 - 15		−3 - 15	−6 - 15		−3 - 15	−6 - 15		−3 - 15	−6 - 15		−6 - 15	−3 - 15		−2 - 15	−6 - 15	
(NA)	3		3	0		2	1		3	0		2	1		0	3	

SD, Standard Deviation; Q1, Quartile 1; Q3, Quartile 3

^1^ Wilcoxon signed rank test for one sample;

^2^ Wilcoxon rank sum test for two independent samples

(a) The test was not performed for HIVTSQs and respective subscales)

(NA) Number of patients for whom no data was available

Table 3 also shows that satisfaction with a blister (measured with HIVTSQc) is not predicted by gender, age group, education level, nationality, or ART duration. On the other hand, a higher baseline treatment satisfaction score was associated with a decrease in the treatment satisfaction change score (six months after the switch to blister). For the HIVTSQc scale model, a one-unit increase in the baseline treatment satisfaction score (HIVTSQs) results in a decrease of 6% [exp (0.06)] in the HIVTSQc overall score.

**Table 3:** Satisfaction change (HIVTSQc scores): Predictive effect of satisfaction at baseline (HIVTSQs), sociodemografic variables, and clinical variables (linear models).

	HIVTSQc scale				HIVTSQc General Satisfaction/ Clinical subscale				HIVTSQc Lifestyle/Ease subscale			
	Model 1		Model 2		Model 1		Model 2		Model 1		Model 2	
	b (95% CI)	p	b (95% CI)	p	b (95% CI)	p	b (95% CI)	p	b (95% CI)	p	b (95% CI)	p
HIVTSQs*	−0.06 (−0.10; −0.02)	**0.002**	−0.06 (−0.10; −0.02)	**0.003**	−0.10 (−0.17; −0.03)	**0.006**	−0.09 (−0.17; −0.02)	**0.016**	−0.09 (−0.15; −0.04)	**0.001**	−0.10 (−0.15; −0.04)	**<0.001**
**Sex**												
Female	—	0.926	—	0.769	—	0.252	—	0.240	—	0.302	—	0.559
Male	−0.03 (−0.56; 0.51)		0.08 (−0.47; 0.64)		0.27 (−0.20; 0.74)		0.29 (−0.20; 0.78)		−0.25 (−0.73; 0.23)		−0.14 (−0.64; 0.35)	
**Age group**												
Les than 50 years	—	0.591	—	0.913	—	0.918	—	0.914	—	0.178	—	0.409
50 years or more	0.12 (−0.32; 0.56)		0.03 (−0.44; 0.49)		0.02 (−0.37; 0.41)		0.02 (−0.39; 0.44)		0.26 (−0.12; 0.65)		0.17 (−0.23; 0.57)	
**Education**												
Secondary education or less	—	0.114	—	0.183	—	0.498	—	0.390	—	0.060	—	0.174
Superior education	−0.34 (−0.76; 0.08)		−0.31 (−0.76; 0.15)		−0.13 (−0.51; 0.25)		−0.17 (−0.57; 0.23)		−0.36 (−0.73; 0.01)		−0.27 (−0.67; 0.12)	
**Nationality**												
Portuguese	—	0.655	—	0.735	—	0.388	—	0.420	—	0.435	—	0.549
Non-Portuguese	−0.10 (−0.52; 0.33)		−0.07 (−0.50; 0.35)		−0.16 (−0.54; 0.21)		−0.16 (−0.54; 0.23)		−0.15 (−0.52; 0.23)		−0.11 (−0.49; 0.26)	
**Antiretroviral therapy duration**												
Up to 5 years	—	0.331	—	0.541	—	0.670	—	0.705	—	0.207	—	0.574
6 years or more	0.21 (−0.21; 0.62)		0.14 (−0.31; 0.59)		−0.08 (−0.45; 0.29)		−0.08 (−0.48; 0.32)		0.24 (−0.13; 0.61)		0.11 (−0.28; 0.51)	

CI, Confidence Interval.

* These coefficients report to scores in HIVTSQs scale, HIVTSQs General Satisfaction/ Clinical subscale and HIVTSQs Lifestyle/Ease subscale in models for HIVTSQc scale, HIVTSQc General Satisfaction/ Clinical subscale and HIVTSQc Lifestyle/Ease subscale, respectively. For analysis, HIVTSQc scores were shifted, reversed and log transformed.

Model 1, Individual models adjusted for each variable presented, plus the HIVTSQs score.

Model 2, Model adjusted for all the variables presented.

This predictive value of baseline satisfaction (HIVTSQs) was also observed for the HIVTSQc subscale (general satisfaction/clinical subscale).

**Fig 3:**
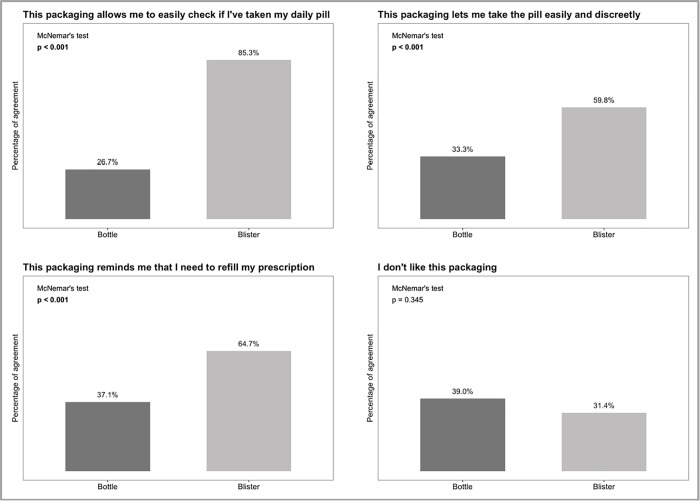
Frequencies of item response on the specifically developed questionnaire between packaging methods.

As can be seen in the plots included in Fig 3, although no significant difference was found in the proportion of patients reporting appreciating positively the packaging (bottle and blister), three main facets of usage were found to be better considered by patients: a significantly higher proportion of them considered that the blister helps them to keep track of daily intake of pills; a higher proportion indicate that blister packaging usage is more discrete than bottle usage; and a higher proportion report that blister packaging helps them to recall the refill momentum.

**Fig 4:**
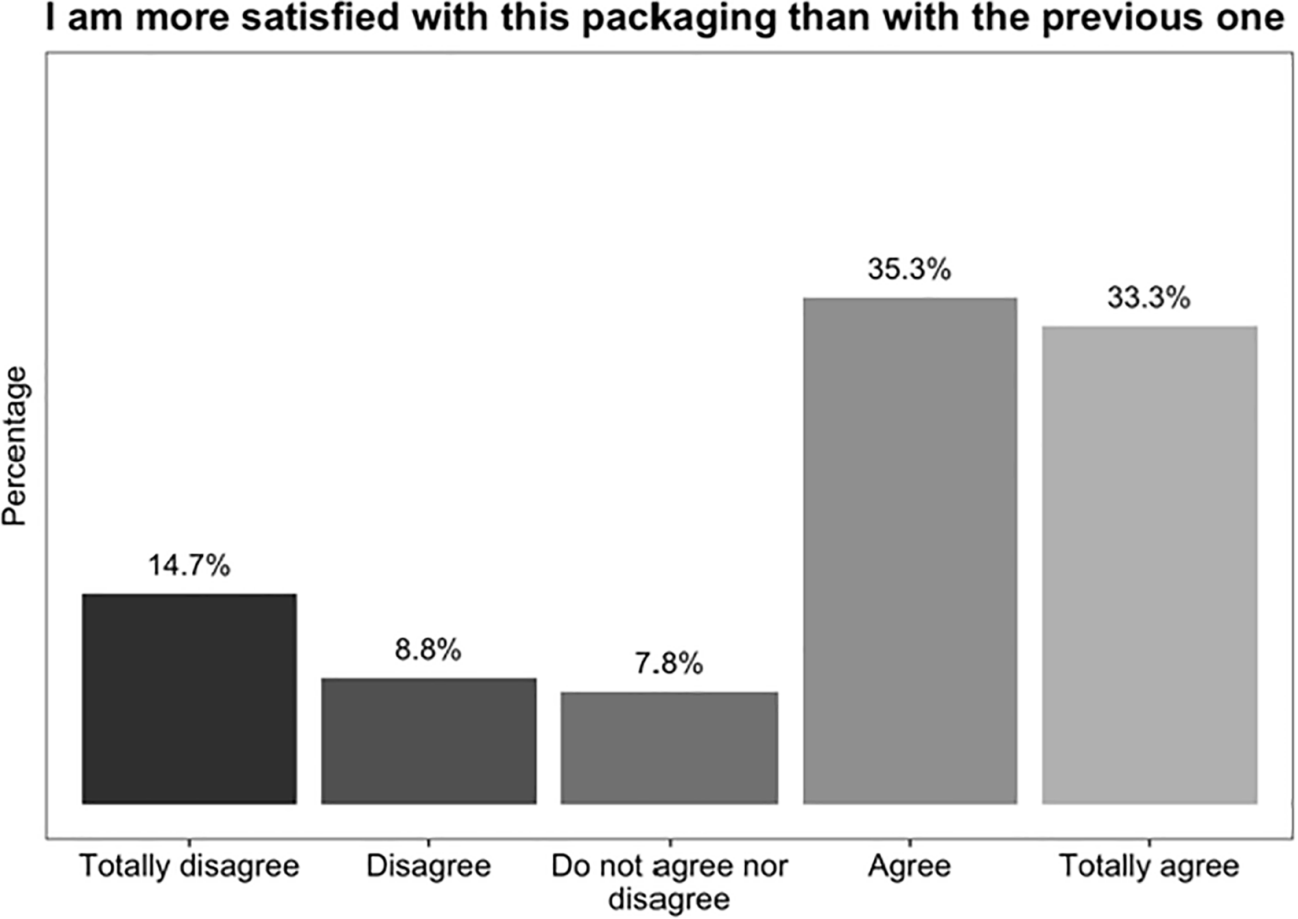
Response distribution on patients’ satisfaction with blister packaging, when compared with bottle packaging.

Overall, and as seen in Fig 4, 68.6% of patients report being more satisfied with the blister packaging than with bottle packaging.

Regarding adherence, no significant differences were observed between the standard pill bottle and monthly blister packaging: 89.7% and 91.3% of adherence, respectively.

## Discussion

The primary objective of this analysis was to assess the impact of introducing blister packaging of Biktarvy^®^ on treatment satisfaction among PLHIV. The transition from bottles to blisters was associated with significant increase in treatment satisfaction, while maintaining an already high level of adherence over the six-month follow-up period. And this increase in satisfaction was independent of several main sociodemographic and clinical characteristics. To our knowledge, this is the first study to evaluate treatment satisfaction with the use of a calendar blister packaging for ART among PLHIV, and it highlights the potential benefits of packaging innovations in enhancing the treatment experience for patients. Importantly, the greatest gains in satisfaction were observed in patients who had been least satisfied with the prior bottle packaging, suggesting that the blister format addressed some unmet needs in this subgroup.

Several factors likely explain why the blister packs improved satisfaction. The design of the blister (weekly calendar-labeled sheets of pills) makes it easier for patients to track daily medication intake and refills. In our study, 85% of patients reported that the blister helped them keep track of whether they took their daily pill, and about two-thirds said it reminded them when to refill their prescription – significantly higher proportions than for the bottle packaging. These findings align with prior evidence that calendar blister packs can serve as effective “drug reminder” packaging, facilitating medication organization and self-monitoring of doses [12]. By providing a visual calendar and one-per-day pill slots, blister packs act similarly to pill organizers, helping to prevent missed doses due to forgetfulness. Indeed, packaging interventions like calendar blisters have been shown to improve adherence in other chronic conditions; for example, a randomized trial in hypertension found that patients using a daily calendar blister refilled prescriptions on time more often and had higher medication possession ratios than those using standard bottles [7]. Also, a meta-analysis with 52 studies across various diseases, likewise showed that switching from pill bottles to calendar blister packs increased adherence from about 63% to 71% on average [8]. Our results suggest that even in a population with good baseline adherence, blister packaging’s convenience and visual cues provided an extra boost to patients’ confidence and satisfaction with managing therapy. Another key contributor to higher satisfaction appears to be the blister pack’s discretion and portability. The blister pack is flatter and can be carried more subtly than a rattling pill bottle with a conspicuous label. Many PLHIV face HIV-related stigma and value privacy regarding their medication. Studies have documented that patients often repackage ART into unmarked containers or bags to avoid unwanted disclosure of their status [13]. This self-repackaging behavior – motivated by the visual identification, bulkiness, and noise of standard pill bottles – has been associated with nonadherence and suboptimal outcomes [13]. In line with this idea, a recent survey of people with HIV indicated that packaging features like ease of use, discretion, and dose tracking are highly valued for supporting adherence [14]. The blister pack’s practical advantages (easy daily tracking), combined with psychosocial benefits (enhanced privacy), provide a plausible explanation for the higher satisfaction observed.

With regard to adherence, we found no statistically significant difference between the bottle and blister periods – adherence remained very high in both (89.7%, at baseline vs 91.3% at endline). There was a modest increase of about 1.6 percentage points with the blister, but this improvement did not reach significance. One reason for the minimal change is the ceiling effect: baseline adherence percentage leaving little room for improvement. In well-managed ART populations, average adherence is typically on the order of 85–90% [12]. Our cohort was at the upper end of adherence even before the packaging change. In such a context, even useful interventions might only yield small absolute gains. This is consistent with prior packaging studies – they tend to show the largest adherence improvements when baseline adherence is low or moderate. For example, a meta-analysis achieved an 8% increase starting from around 63% adherence [8], whereas in our high-adherence group the increase was only around 2%. It is important to note, however, that maintaining higher than 90% adherence with the blister over six months is itself a positive outcome, indicating that the new packaging did not jeopardize adherence and possibly helped prevent any decline in adherence over time. Longer follow-up might reveal whether the small uptick in pharmacy refill adherence with blisters could translate into significant clinical benefits (e.g., sustained viral suppression rates) or whether adherence improvements emerge in subpopulations with initially lower adherence.

Another main finding regards the perception of utility from the blister packaging: after a minimum of six month of experience, the blister (which includes a weekly calendar) was considered by the majority (85%) of patients as an effective tool to keep track of their daily intake of pills (significant higher percentage than the appreciation for bottle packaging), and about two third of patients considered that it is helpful as a reminder of the adequate moment of refilling.

This study has limitations that should be acknowledged. First, the sample size was relatively small and limited to two hospitals, which may affect the generalizability of the findings. It is, nevertheless, worth noting that power calculations indicated the study had enough power (with 95% confidence) to detect change in satisfaction and adherence. Second, the representativeness of different subpopulations of PLHIV was not fully captured, potentially limiting the applicability of the results to more diverse patient groups, requiring additional research. In particular, certain subpopulations of PLHIV (such as women, younger patients, or those from other regions) were under-represented, so their specific packaging preferences and outcomes may not be fully captured. Third, the follow-up period after the introduction of the blister packaging was relatively short (approximately six months). This short timeframe might not have been sufficient to observe long-term changes in adherence behavior or clinical outcomes like viral load suppression. It also prevented us from assessing whether the initial boost in satisfaction is sustained over time or if any novelty effect wears off. Finally, the study’s observational pre-post design (without a concurrent control group continuing on bottle packaging) means we cannot completely rule out other temporal factors or biases that might have influenced satisfaction and adherence (e.g., patients’ growing familiarity with Biktarvy^®^ or other aspects of care over the study period).

## Conclusion

Our findings underscore that seemingly simple changes in how medication is packaged can have meaningful effects on patient experience. Packaging should be considered as part of ART management strategies, alongside drug selection and counseling. By enhancing ease-of-use and privacy, optimized packaging might encourage patients to remain engaged in care and adhere to therapy. The blister pack innovation here studied is a relatively low-cost intervention that required no change in medication or dosing, yielding a clear improvement in patient-reported satisfaction. Our study adds to the growing recognition that “*beyond the pill*” factors (such as packaging and delivery mechanisms) can impact treatment success in HIV care. Future research and implementation projects could explore integrating calendar blister packs into routine ART distribution, and evaluate outcomes like adherence, persistence in care, and patient satisfaction with larger and more diverse populations, and over longer follow-up periods.
